# Prostate Microbiota and Prostate Cancer: A New Trend in Treatment

**DOI:** 10.3389/fonc.2021.805459

**Published:** 2021-12-10

**Authors:** Bangwei Che, Wenjun Zhang, Shenghan Xu, Jingju Yin, Jun He, Tao Huang, Wei Li, Ying Yu, Kaifa Tang

**Affiliations:** ^1^ Department of Urology, The Affiliated Hospital of Guizhou Medical University, Guiyang, China; ^2^ Department of Stomatology, The First Affiliated Hospital of Fujian Medical University, Fuzhou, China; ^3^ Institute of Medical Science of Guizhou Medical University, Guiyang, China

**Keywords:** prostate cancer, tumor microenvironment, microbiota, therapy, inflammation

## Abstract

Although the incidence and mortality of prostate cancer have gradually begun to decline in the past few years, it is still one of the leading causes of death from malignant tumors in the world. The occurrence and development of prostate cancer are affected by race, family history, microenvironment, and other factors. In recent decades, more and more studies have confirmed that prostate microflora in the tumor microenvironment may play an important role in the occurrence, development, and prognosis of prostate cancer. Microorganisms or their metabolites may affect the occurrence and metastasis of cancer cells or regulate anti-cancer immune surveillance. In addition, the use of tumor microenvironment bacteria in interventional targeting therapy of tumors also shows a unique advantage. In this review, we introduce the pathway of microbiota into prostate cancer, focusing on the mechanism of microorganisms in tumorigenesis and development, as well as the prospect and significance of microorganisms as tumor biomarkers and tumor prevention and treatment.

## Introduction

Prostate cancer is the most common urinary system tumor in men, and its biological characteristics lead to late clinical diagnosis. The 5-year survival rate for prostate cancer is close to 100 percent, thanks to breakthroughs in early identification and treatment made possible by increasing prostate cancer screening (1). However, around one-third of patients will inevitably suffer biochemical recurrence/progression, eventually progressing to metastatic disease (2). Although conventional treatments like as surgery, chemotherapy, radiation, and androgen deprivation therapy have improved overall survival rates in these individuals, the 5-year survival rate remains about 30% (3, 4). In recent years, the direct or indirect relationship between microorganisms and tumors has been continuously studied and reported. There is an extensive relationship between cancer and specific microflora of different cancer types (5). It is not only reflected in the correlation of specific microflora in slowing down tumor growth or promoting tumor metastasis (6), but also in the use of microbial targeting to treat tumors (7).

The relationship between prostate microflora and prostate cancer has always been a direction worthy of consideration. The increased risk of cancer caused by changes in microbiota has been confirmed in other tumors (8). The correlation between prostate microbiota and the occurrence and metastasis of prostate cancer has also been preliminarily reported (9, 10). Although there is a lack of in-depth studies to explain the mechanism of its involvement in the disease process of prostate cancer, it is undeniable that prostate microbiota may be an important breakthrough to explore the pathogenesis of prostate cancer, new treatment strategies, and improve the prognosis of patients.

In this review, we summarize previous studies on prostate cancer microbiota to clarify the potential role of prostate microflora in the occurrence and development of prostate cancer and new strategies for the prevention and treatment of prostate cancer based on microbiota.

## Gut Microbiota and Prostate Cancer

The gut microflora is the largest bacterial bank in the human body. Changes in the composition of gut microflora and/or biological imbalances may have an impact on the occurrence and development of tumors (11). As a result, nearly all past research has concentrated on prostate cancer and gut bacteria. Previous research has demonstrated that the gut microorganisms of prostate cancer patients differ from those of healthy or benign prostate disease patients, with a higher relative abundance of Bacteroides massiliensis in prostate cancer patients’ intestines. Faecalibacterium prausnitzii, on the other hand, has a lower relative abundance (12). Acetic acid can be metabolized by Faecalibacterium prausnitzii to butyric acid, the most prevalent short-chain fatty acid in the colon (13). It possesses anti-tumor activities, mostly through inducing apoptosis and decreasing proliferation (14), as well as enhancing cell differentiation and mechanically inhibiting histone deacetylase in cancer cells (15). Furthermore, a study of fecal microbiota revealed a significant difference in the abundance of bacteria and streptococci between prostate cancer and non-prostate cancer, with the metabolic pathways related to folic acid and arginine being the most significant (16). Folic acid is essential for nucleotide synthesis and DNA methylation. Folic acid deficiency causes DNA instability and a high mutation rate (17). According to one study, folic acid-producing microflora is more abundant in non-cancer patients than in cancer patients, implying that natural folic acid sources may protect against prostate cancer (18). In other words, gut microbes and their metabolites have an effect on the occurrence and progression of prostate cancer. By modifying the composition of gut microbes, it is possible to prevent and treat prostate cancer.

## Prostatic Microorganisms and Their Potential Sources

**Figure 1:**
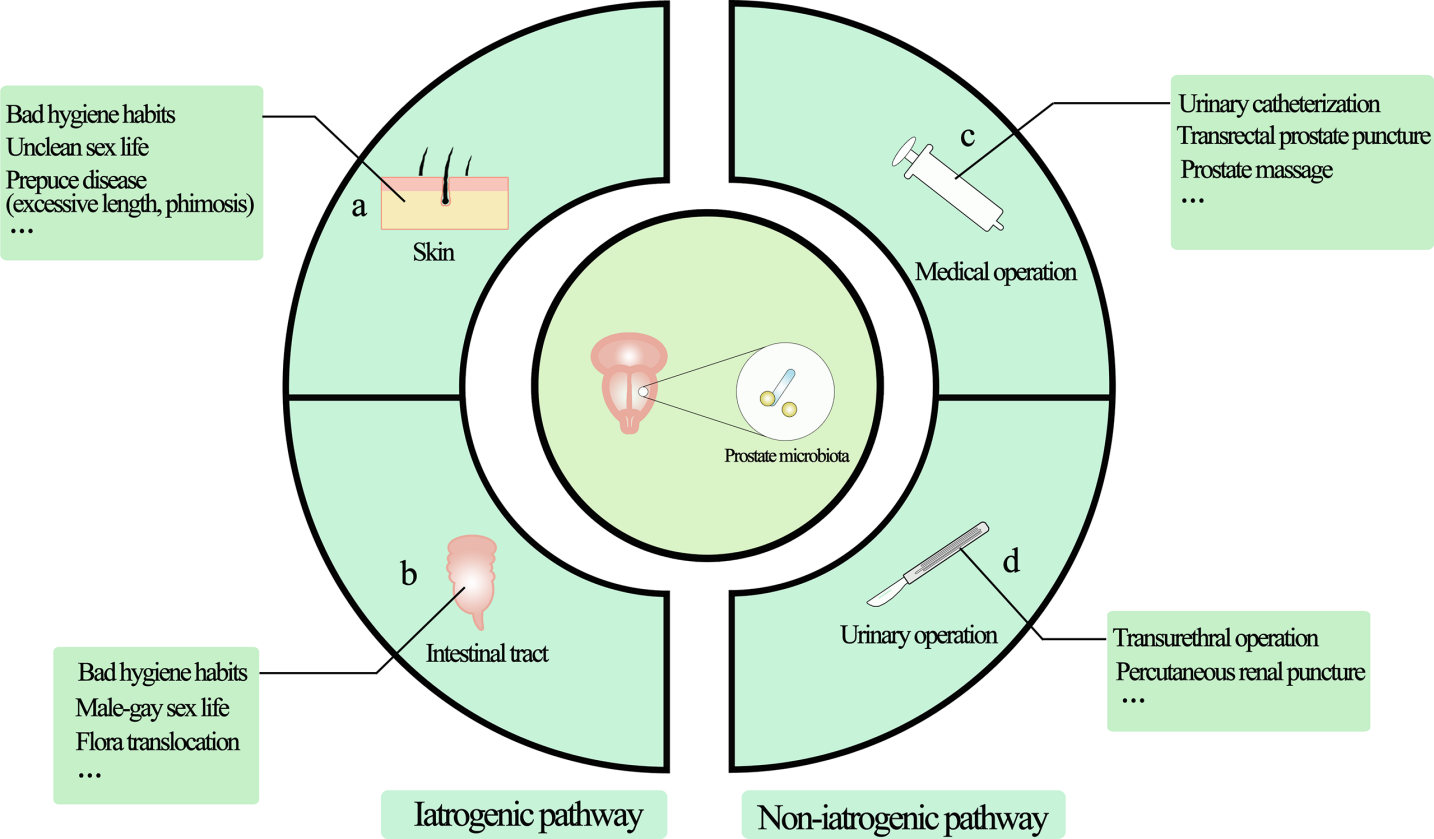
The main source of prostate microorganisms. The source of prostate microorganisms is mainly from the external environment of microorganisms through the urethra into the urinary system and implanted in the prostate due to various factors, mainly divided into iatrogenic pathway and non-iatrogenic pathway. **(A)** Skin-derived microbial infections are mainly microorganisms located in the vulva or other parts through the urethral orifice with the help of bad/unclean living habits (including sexual life), such as Propionibacterium acne and staphylococci. **(B)** Intestinal-derived bacteria such as Escherichia coli are the most common bacteria in UTIs, so they are also the most common bacteria in the prostate, mainly from retrograde infections. **(C)** Non-standard medical procedures may bring microorganisms from the external environment into the urethra through instrument consumables to cause microbial colonization. **(D)** Patients who need urinary tract surgery often have microorganisms in their urinary system, and microorganisms can be transferred with the help of surgical procedures. Non-standard surgical procedures can also bring microbes from the external environment into the urinary tract system.

Obviously, the genitourinary tract is not a sterile environment (19). Due to normal voiding activity, making most of the time in an asymptomatic state. Over the last few decades, a number of studies have found evidence of the presence of microbes in tissues with prostate diseases, including bacteria (20–22) and viruses (10, 23, 24), which may result from bacterial translocation such as the skin and intestines or infections through high-risk sexual behavior (**Figure 1**) (25, 26). Given that most studies used a variety of techniques, such as culture, confocal microscopy for microorganism visualization, and analysis of bacterial 16Sr RNA gene using environmental hybridization, immunohistochemistry, and PCR, the results show that the composition of prostate microflora is similar to that of urethra (20, 27–29). The main pathogen of urinary tract infection (UTI) is Escherichia coli, represented by Gram-negative bacteria, and it is generally believed that the main way is retrograde infection, that is, because there is no valve protection in the urethra, some bad living habits (hand hygiene, high-risk sexual behavior) allow them to enter the urethra through the urethral orifice and then colonize in the prostate. In addition, there may be other ways for microorganisms to enter the urethra, that is, intestinal bacterial translocation. The process by which bacteria and their products migrate from the gastrointestinal tract to the blood and other organs is called “bacterial translocation”, which was first described in the 1960s (30). The risk factors for bacterial translocation primarily comprise three aspects: intestinal microflora imbalance, damage or increased permeability of the intestinal epithelial barrier, and host health status. Although so far, no direct studies have shown that bacteria can reach the prostate from the intestinal tract through Bacterial translocation, this guess is possible. Some Escherichia coli in the intestinal tract have the ability to adhere to and invade intestinal epithelial cells and survive in macrophages. These Escherichia coli can reach the lamina propria of the intestinal tract through paracellular or cross-cellular pathways (31–33). Macrophages may then transfer bacteria from the intestines to lymph nodes, systemic circulation, or even distant organs to produce inflammation (34, 35). This may also explain why some clinical studies have found that the same kind of E. coli has been detected in the intestines, blood, and urine (36, 37). Although the translocation of bacteria mainly occurs in people with low immunity, it also occurs as a normal phenomenon in healthy people, and most of them do not have obvious clinical manifestations, with a translocation rate of about 5%-10% (38). These results suggest that under natural conditions, the source of prostate microflora may be mainly affected by the external microenvironment, especially the urinary tract microflora and intestinal microflora.

Second, iatrogenic UTI, particularly catheter-related infections, is a significant source of prostate microbiota (25). The use of a urethral catheter or urethral stent is a significant risk factor for UTI, and the incidence of bacteriuria linked with it can increase by 3% to 8% every day (39). Studies have shown that bacteria entering the urinary tract can be colonized by bacteria in distant organs through catheters and indicate the formation of biofilms in the mucous membranes of the organs, such as the prostate (40, 41). Although studies have shown that the most common microorganism of catheter-related infection is Escherichia coli, it is often found that some drug-resistant bacteria can also enter the genitourinary tract through the catheter (42). Therefore, it is not surprising that bacteria in biofilms tend to be highly resistant to antibiotics and host immune defense mechanisms. In addition, biofilms have complex components, including not only microorganisms, but also a variety of major biological macromolecules, such as proteins, polysaccharides, DNA, RNA, peptidoglycan, lipids, and phospholipids, which can produce a variety of complex reaction mechanisms to invade cells and tissues (43, 44). In short, iatrogenic catheter-related microorganisms can overcome the host defense mechanism, regulate the host immune response through a variety of mechanisms, and help escape the host immune system, establish or aggravate bacterial infection, and cause persistent and chronic inflammation.

However, not all microbes can cause bacterial colonization of the prostate after entering the urinary tract, because the human body has a certain ability of self-defense against the invasion of bacteria into the urinary tract (45). When the urinary tract is unobstructed, the urine can wash away most of the bacteria (46). At the end of urination, the prostatic fluid excreted from the posterior urethra has a certain killing effect on bacteria, which may be due to the high concentration of zinc ions in the prostatic fluid. It has been proved to have bactericidal effect on a variety of Gram-positive and Gram-negative bacteria *in vitro (* 47, 48). Urinary tract mucosa can play a bactericidal effect by secreting organic acids, IgG, IgA, and phagocytes (49). Under normal circumstances, the pH value of urine is low, contains high concentrations of urea and organic acids, and the urine itself is too low or high tension, which is not conducive to bacterial growth (50). Therefore, microorganisms are easy to colonize and grow only when there is urinary tract obstruction, injury, deformity, and decreased body resistance.

## Effects of Drug Therapy and Dietary Therapy on Prostatic Microorganisms

According to the guess of the possible origin of the prostate microflora (urethra and intestinal tract), it is undeniable that drugs and diet may also have an effect on prostate microbes. Previous studies have shown that some drugs or diets can alter the structure of urethral and gut bacteria (51, 52). The microflora in the human body activates a variety of stress mechanisms in response to antibiotic therapy, including genomic mutations/modifications and the production of enzymes to degrade antibiotics (53). Long-term use of antibiotics will interfere with the activities of normal bacteria, thus changing the structure of microbial communities. When the microbial community structure of the urethra or intestines changes, the prostate microflora may also change. In addition, there are differences in the ability of different drugs to permeate prostate tissue. In a study of quinolones, it was found that the ability of the drug to penetrate into the prostate tissue was norfloxacin < fluidixacin < ciprofloxacin < ofloxacin < fleroxacin (54). Different quinolones also have differences in the types of microorganisms that can act, resulting in differences in the structure of the bacteria. The effect of diet on prostatic bacteria also depends to a large extent on the regulation of gut bacteria (55). Previous studies have shown that the structural types dominated by Bacteroides and Bifidobacterium are positively correlated with high-fat diet, high animal protein, amino acid and saturated fat intake, and negatively correlated with fiber intake. The main structural type of Prevotti is related to the high consumption of carbohydrates and monosaccharides (56). In addition, animal studies have shown that a long-term high-salt diet is not only a risk of high blood pressure, but also leads to a reduction in the number of Bacteroides and Proteus in the intestinal tract (57). Although there is a lack of direct research on the effects of drugs or diet on the microbial composition of the prostate, this will be clear as the concern of the prostate microbiota increases. In the future, drug therapy (antibiotics)/diet therapy may prevent and treat prostate cancer by regulating the microflora.

## Prostate Microflora and Prostate Cancer

Microbes have been shown to be involved in the process of prostate disease (58–60). Normal prostate tissue contains a variety of immune cells, including lymphocytes in the stroma or epithelium (61). So, regulating the immune process may be one way in which microorganisms participate in affecting tumor development (62, 63). Although the exact mechanism of this approach in driving the transformation of prostate cells into tumors is unclear, there is sufficient evidence to link it to the potential role of microorganisms and their metabolites, which may directly lead to prostate cell genetic instability. This leads to abnormal cell proliferation and tumor development (64–66). In addition, microorganisms in the tumor microenvironment also seem to regulate the apoptosis of prostate cancer through a variety of mechanisms (60, 67) (**Table 1**).

**Table 1:** Study on the pathways related to the carcinogenesis of some microorganisms.

Microbial species		stimulus	Cellular Target	Mechanism/Effect	Refs
Bacteria	Escherichia coli	LPS, CNF1	NF-κB, Cdc42, TLR	Promote value-added, Promote distant metastasis, inhibit apoptosis,	(66, 68, 69)
	Propionibacterium acne	PG	VEGF, NF-κB, MAPK, cGAS-STING	Increased inflammation	(70, 71)
	staphylococcus	SEH	lncRNAs	Promote apoptosis of tumor cells	(72)
	Chlamydia trachomatis	Intracellular parasitism	IL-6, FGF-2, VEGF, ICAM-1, NF-κB	Progress, transfer, Increased inflammation	(73, 74)
Virus	HPV	CpG DNA, E2, E6, E7	NF-κB, TLR, P53, Rb, Bcl-2, survivin, E-cadherin, N-cadherin, Twist, PTPN13 and SLUG	Promote value-added, transfer inhibit apoptosis,	(68, 75, 76)
	HSV	CpG DNA,	NF-κB, TLR,	Promote value-added, inhibit apoptosis	(68)
	MRV	VAP	RACK1, caspase8	Induce apoptosis of tumor cells	(77)
	BKV	LT Ag	P53	Promote growth	(78)

LPS, Lipopolysaccharide; CNF1, Cytotoxic Necrotizing Factor 1; PG, Peptidoglycan; SHE, Staphylococcal Enterotoxin H; VAP, Virus Attachment Protein.

## Inflammatory Pathway of Prostate Microbiota (Indirect Pathway)

**Figure 2:**
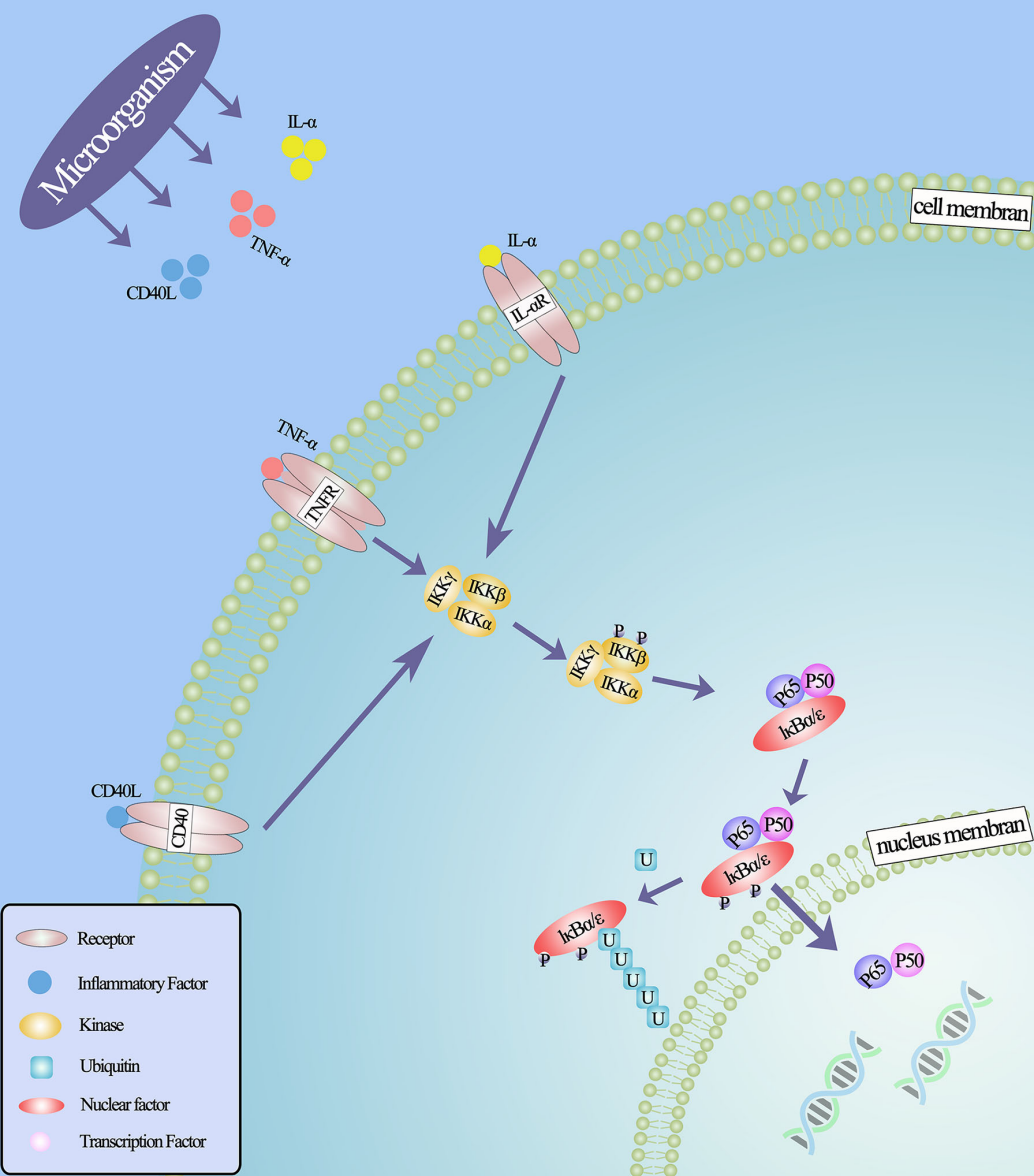
NF-κB classical signal pathway under the action of microorganisms. Microorganisms stimulate inflammatory cells to secrete inflammatory factors that bind to related receptors and cause configuration changes, such as IL- α (IL-6, IL-8), TNF- α, CD40, lipopolysaccharide and so on, thus activating IκB kinase. This leads to the phosphorylation and ubiquitin of IκB protein, the degradation of IκB protein and the release of NF-κB dimer. Through various post-translational modifications, NF-κB dimer is further activated and transferred to the nucleus to bind the target gene and promote the transcription of the target gene.

Inflammation is generally thought to be associated with malignant tumors (79), and little is known about how these microbes promote prostate cancer in the prostate microenvironment. However, from previous studies, it has been found that microbes implanted in the prostate may promote tumorigenesis by inducing chronic inflammation and related immune responses. It is well known that in the process of chronic inflammation, microorganisms can induce and regulate the expression of various cytokines and chemokines in inflammatory cells (80). These cytokines, secreted in the prostate microenvironment, can further regulate a variety of mechanisms. For example, inflammatory factors IL-6, IL-8 and TNF-α can induce the expression of vascular endothelial growth factor (VEGF) and activate NF- κB, EGFR, TLR and other signal pathways, thus stimulating tumor cell proliferation. These pathways have carcinogenic and anti-apoptotic activities (73, 81–85). Because the NF- κB signaling pathway is widely used by eukaryotic cells as a genetic regulator to control cell proliferation and cell survival, it may be the key to microbial induction of prostate cancer (**Figure 2**). Through *in vitro* studies, it is found that in the early stages of inflammation, inflammatory factors will increase due to the stimulation of microorganisms and their metabolites (86). And the transcriptional activities of p65 and IκBα genes in infected prostate epithelial cells were significantly higher than those in uninfected cells, indicating that the NF-κB signaling pathway was involved in the occurrence of prostate cancer in the early stage of inflammation, and the activation of NF-κB was time-dependent with the stimulation of inflammation (68, 73). In an animal experiment, mice infected with polyomavirus developed prostatic intraepithelial neoplasia on the ventral and dorsal sides of the prostate, and transcriptional map analysis showed regulation of multiple pathways, including NF-κB (87). In other words, the use of some means to regulate the activity of NF-κB may lead to the loss of the cancerous potential of prostate cells.

In addition, in the previous study, using the mouse prostatitis model established by Escherichia coli, it was found that there were different degrees of atypical and dysplastic prostate tissue, and the DNA oxidative damage and epithelial cell proliferation in these abnormal tissues were stronger than those in normal prostate tissue. This suggests that specific bacteria can induce prostatic hyperplastic inflammatory atrophy (PIA) and prostatic intraepithelial neoplasia (PIN) through inflammation and oxidative stress (88). PIA and PIN are considered to be the precursors of prostate cancer (89). At present, it is believed that reactive oxygen species (ROS) and reactive nitrogen (RNS) released by inflammatory cells repeatedly cause oxidative stress damage to normal prostate tissue, and the reactive changes of prostate cells occur in the process of continuous injury and regeneration. In addition, chronic inflammation caused by bacterial infection in the microenvironment can not only lead to the occurrence of PIA and PIN, but also accelerate the PIN process by activating macrophages. Further studies also found that in the region where PIN occurs, PIN cells can recruit macrophages to gather by secreting ICAM-1 and CCL2, while recruited macrophages can activate ERK and JNK signals in PIN cells by secreting C5a, CXCL1, and CCL2 to promote the proliferation of PIN cells (90). Therefore, chronic inflammation of the prostate caused by bacteria may play a role in initiating the progression of prostate cancer.

## Direct Pathway of Prostatic Microbiota

### Typical Bacteria

Specific bacteria can participate in the occurrence and development of prostate cancer by producing toxins. For example, lipopolysaccharide (LPS) is the main component of the cell wall of bacteria such as Escherichia coli and Neisseria gonorrhoeae. It belongs to a bacterial endotoxin and is released after bacterial cleavage. It has been found that there is a significant relationship between LPS and prostate invasiveness (91). When continuously activated by bacterial LPS, a series of closely related genes such as cell proliferation, differentiation, and apoptosis are abnormally overexpressed, including stimulating several pathways downstream of TLR4, namely IL-6/STAT3, AKT/GSK-3β, and β-catenin pathways, to induce epithelial-mesenchymal transformation (EMT) of prostate cells (92). In addition to endotoxins, exotoxins secreted by bacteria can also promote the progression of prostate cancer. Toxic necrosis factor (CNF1) can accelerate the progression of prostate cancer by activating the Cdc42-PAK1 signal axis (69). Additionally, in some *in vivo* and *in vitro* studies, it has been found that some bacterial toxins show anti-tumor properties, such as alpha toxins (93) and enterotoxins (94–96). In these studies, it is shown that bacterial toxins can regulate the apoptosis of tumor cells through a variety of mechanisms. Enterotoxin expressed by Staphylococcus aureus can induce apoptosis of PC3 cells, which involves changes in the expression of lncRNAs, including Gas5, PCA3 and NEAT1 genes (72). Botulinum toxin A may enter the cell through the SV2 receptor of neurotoxin and induce phospholipase A2 (PLA2) phosphorylation to inhibit the growth and proliferation of prostate cancer cells (97). Thus, it can be seen that bacterial toxins have great potential in the treatment of cancer. However, the mechanism of bacterial toxins in the occurrence and development of human prostate cancer has not been fully elucidated. More research is needed to explore its potential molecular mechanisms in order to find more effective cancer prevention and treatment strategies.

### Mycoplasma

Mycoplasma is one of the most common atypical bacteria. Mycoplasma infection may play an important role in the pathogenesis of prostate cancer, probably because it itself and its proteins are common components of the tumor microenvironment (98). The effect of mycoplasma on prostate cancer is mainly realized by its protein. Balanced subcellular localization (BaCeILo) is a predictor of the subcellular localization of eukaryotic proteins (99). In the past, this system has been used to predict the subcellular localization of mycoplasma hominis proteins in different parts of the host, including 320 cytoplasmic proteins, 77 mitochondrial proteins, 29 nuclear proteins, and 137 secretory proteins. These proteins may have potential effects on prostate cancer (100). The chaperone protein DnaK of mycoplasma has carcinogenic activity by binding to poly (ADP-ribose transferase) and p53, thus inhibiting DNA repair and p53 function (101). Exogenous DNAK in the tumor microenvironment can activate some kinase-related transduction pathways in a cell-specific way, such as the Akt1/2/3 group and p70S6 kinase group of the AGC family, the AMPKERK2 group of the CAMK family, and α-1-2-3 group of the CMGC family, so as to promote carcinogenesis and cancer progression (102). In addition, the protein p37 encoded by mycoplasma can promote the invasion of cancer cells in a dose-dependent manner (103). *In vitro*, p37 can not only induce the expression of inflammatory cytokines, but also activate multiple signal pathways by activating protein kinases, including the phosphatidylinositol 3-kinase (PI3K)/AKT cascade, protein kinase C (PKC) family and mitogen-activated protein kinase (MAPK)/RAS signal cascade, which increases the invasiveness of prostate cancer (104, 105). However, there is little literature on mycoplasma and prostate cancer, and more research on mycoplasma protein in the future may help to understand its role in prostate cancer in the tumor microenvironment.

### Virus

The prostate microflora contains many specific viruses, which have a potential pathogenic mechanism in the process of prostate cancer. In the past few decades, studies on human papillomavirus (HPV), adenovirus, BK polyomavirus (BKPyV) and EB viruses have preliminarily shown the possible carcinogenic and metastatic mechanisms of the viruses in the prostate (23, 87, 106, 107).

More and more studies suggest that there is a close link between the virus and prostate cancer. Recent studies have suggested that early detection of the virus may help reduce the risk of prostate cancer caused by the virus (108, 109). Previous studies have found that the prostate may be the site of HPV replication (110), and most studies have shown that HPV is closely related to prostate cancer (111, 112). Studies have shown that the incidence of high-risk HPV in benign prostate tumors is similar to that found in prostate cancer (112). At the same time, after a systematic review of 26 studies, it was found that the proportion of high-risk HPV in prostate cancer was significantly higher than that in benign tumors and healthy prostates (113). In addition, recent case-control studies supported this view, which found that the average expression levels of inflammatory mediators (IL-17, IL-6, TNF-α, NF- κB, VEGF, ROS, and RNS), anti-nesting factors (N-cadherin, slug, and twist) and anti-apoptotic mediators (Bcl2 and Survivin) were significantly increased in HPV positive samples. The average expression levels of tumor suppressor proteins (p53 and pRb) and E-cadherin (inhibitor of apoptosis) were significantly down-regulated, suggesting that HPV infection may participate in the metastasis of prostate cancer by regulating the behavior of prostate cancer cells (75, 84). In addition, some studies have also found that HPV and EBV may interact with each other to promote the survival and proliferation of cancer cells (114). Other functional proteins such as polyomavirus large T antigen (LTag) can interfere with the infected cell cycle by binding to p53, thus inactivating its tumor suppressor function. This inactivation enables infected cells to activate carcinogenic transformation (115, 116). Unlike HPV or adenovirus, BK virus participates in the growth of prostate cancer by isolating p53 into a protein complex (LTAG-p53 complex) in the cytoplasm, thereby disabling its function (117). In addition, the study also reported that there was a significant correlation between a special regulatory feature caused by LTag peptide pool stimulation and evidence of biochemical recurrence in BKPyV-positive prostate cancer patients (63).

Of course, viruses do not always have a positive effect on prostate cancer, although initial studies have shown that adenoviruses have a carcinogenic effect on animals (118). However, with the deepening of research, the results often suggest that some adenoviruses can inhibit the development of prostate cancer, so it is generally considered that it may be one of the breakthroughs in the targeted therapy of prostate cancer (119). Previous studies have found that adenoviruses can produce at least 50 serotypes. Through the study of various serotypes, it is found that serotype 12E1A inhibits AR-mediated transcription and prostate cancer cell survival, suggesting that E1A12-targeted AR may have a potential therapeutic effect on the treatment of advanced prostate cancer with increased AR (120). In addition to its serotype affecting the development of prostate cancer, adenovirus expressing Fas ligand (FasL) can induce apoptosis in a group of prostate cancer cell lines. At the same time, the apoptotic bodies and cell fragments produced by cells infected with this type of adenovirus can continue to induce FasL-mediated apoptosis in uninfected neighboring cells (121). It can be seen that the virus plays an important role in the occurrence and progression of prostate cancer, and increasing research in the field of the virus may be of great significance to the understanding and prevention of prostate cancer.

In short, bacteria and viruses both play positive and negative roles in the formation and progression of prostate cancer, although this complex interaction remains unknown. A more specific characterization of the role of prostate microflora in prostate cancer may open up new avenues for prostate cancer prevention and treatment in the future.

## Effect of Sexually Transmitted Pathogens on Prostate Cancer

Although there are many controversial conclusions about the impact of sexually transmitted diseases on prostate cancer, it is undeniable that they may have potential effects on the susceptibility and recurrence of prostate cancer, including trichomonas vaginalis, HPV, Neisseria gonorrhoeae, HIV, cytomegalovirus, and human herpesvirus (21, 122–125). A prospective study showed that patients diagnosed with prostate cancer had significantly higher than those in the control group in terms of the number of partners, ejaculation frequency, and serum inflammatory parameters (126). The reason for this result may be that high-risk sexual behavior increases the chances of sexually transmitted infections (125, 127, 128). In the case of men having sex with multiple heterosexual partners or having sex with same-sex partners, the results were unexpectedly consistent, with a significantly increased risk of prostate cancer (129).

Trichomoniasis caused by Trichomonas vaginalis infection is the most common sexually transmitted disease. Studies have shown that Trichomonas vaginalis increases the risk of prostate cancer. When Trichomonas vaginalis infection causes inflammation, a variety of cytokines are expressed, such as IL-6, IL-8, KF- κ B. These cytokines will interact with macrophage migration inhibitory factor, PIM-1, and prostate specific antigen (PSA) to polarize macrophages into M2 and induce prostate cancer cell proliferation and migration (130, 131). Another common vaginal microbe is Gardnerella vaginalis. When infected by Gardnerella vaginalis, LPCAT2, TLR3, and TGFB2 genes will be down-regulated or deleted, and the loss of the function of these genes will directly promote the progression of prostate cancer (6, 132). Chlamydia trachomatis is an atypical bacterium that can be transmitted sexually. A recent *in vitro* study showed that Chlamydia trachomatis can proliferate in prostate cancer cells, resulting in enhanced transcription of IL-6 and FGF-2 genes, while FGF-2 can promote vascularization and metastasis of primary prostate cancer (133). In addition, after Chlamydia trachomatis infection, NF- kappa B was activated, TLR2 and TLR4 were significantly up-regulated, which promoted tumor progression (73). Therefore, early popularization of healthy sexual knowledge and safety measures may help to prevent the occurrence of prostate cancer.

## Treatment of Prostate Cancer Based on Microorganism

Early diagnosis of prostate cancer includes PSA testing and rectal biopsy, and treatment includes surgical resection, hormone therapy, chemotherapy, and radiotherapy. However, these methods have some limitations. For example, prostate cancer can occur even if PSA levels are lower than 4.0ng/ml, and this is not uncommon (134). Therefore, it is of great significance to find new auxiliary means or auxiliary methods for diagnosis, treatment, or monitoring. Prostate microbes are almost involved in the whole process of prostate cancer. The combination of microbiology and cancer may bring new breakthroughs.

## Microbial Components as Biomarkers

PSA is a widely used biomarker for prostate cancer. However, due to the pathological characteristics of PSA, the results have some limitations (135). Therefore, more biomarkers are needed as complementary tools for prostate cancer prediction and monitoring. In view of the important relationship between tumors, immunity, and microorganisms, it may help us to find more potential biomarkers in the field of microbiology (136). The increased expression of the human endogenous retrovirus (HERV) sequence is associated with prostate cancer, which suggests that it may be used as a new marker for the diagnosis or prognosis of prostate cancer (137). By detecting the HERV transcripts of matched cancerous and benign tissues in patients with prostate cancer and comparing them with men without prostate cancer, it was found that the high expression of HERV-K Gag was limited to malignant cells, indicating the potential utility of HERV-K Gag as a prostate cancer marker in diagnosis, prognosis, and treatment (138). In addition, unlike tissue biopsies, biofluid analysis is a non-invasive method for screening for prostate cancer. The excretion of urine needs to pass through the prostate and will be mixed with some prostate fluid, so the microRNAs (MiRNAs) of urine microorganisms may become a potential biomarker for the diagnosis and evaluation of prostate cancer. Previous studies have initially confirmed this possibility. HSV1-miR-H18 and HSV2-miR-H9-5p derived from herpes simplex virus (HSV) in urine are superior to serum PSA in detecting the gray area of prostate cancer (109). Interestingly, some studies have shown that with the help of special recombinant viruses, it seems to be able to label circulating tumor cells. That is, it may help to determine whether there is a very early metastasis, which will be greatly conducive to the choice of treatment and prognosis (139). Therefore, it is possible to find more reliable tumor biomarkers in the field of microbiology.

## Microbial Diagnostic Tools

The use of microorganisms as a tool for disease diagnosis is a new field. Biomarkers have always been used as the basis for disease diagnosis, but because of the limitations of their accuracy, the early diagnosis of many diseases is still challenging. The human microbiome, on the other hand, functions like a dynamic recorder, constantly capturing data on physical health, sub-health, and disease status. As a result, detection of human microbiota may make disease diagnosis more reliable, effective, and timely. A new study confirms the feasibility of using the characteristics derived from a group of intestinal microflora to accurately diagnose liver cirrhosis in people with non-alcoholic fatty liver disease (140). This result is based on the fact that key microbial species may play a causal role in the pathophysiology of liver cirrhosis. Another study uses the detection of oral microbiota to make a more detailed classification of halitosis so as to provide a more accurate treatment (141). Unfortunately, there are few studies on microorganisms as diagnostic tools, and there is still a lot of room for development in this field. Perhaps in the future, the structural characteristics of microflora in prostate puncture tissue samples or prostatic fluid may help us diagnose early prostate cancer and predict its malignant degree.

### Microbial Immunotherapy for Prostate Cancer

Tumor microenvironment is typically beneficial to inflammation and immunosuppression, owing to the function of cancer-related fibroblasts in promoting tumor development and an increase in TGF- secreted by prostate tissue, which inhibits the function of NK cells and lymphocytes (142, 143). Through the study of microbial metabolomics, most of the body’s microbes and their metabolites have the ability to stimulate the body to produce immunomodulatory effects. They may treat tumors through mechanisms that affect immune responses through their effects on host immune cells. In addition, it has been reported that prostate tissue-specific microorganisms may improve the immunogenicity of tumors and make drug-resistant cancer types sensitive to immunotherapy (144). In fact, the immunotherapy of microorganisms should not be underestimated.

Therapeutic virus vaccine can activate the immune system, induce specific cellular and humoral immune responses through tumor cells or tumor antigen substances, enhance the anti-cancer ability of the body, and prevent the growth, proliferation and recurrence of tumors, thus achieving the purpose of eliminating or controlling tumors. Recombination of some special viruses into virus vaccines, such as vaccine viruses based on adenovirus, also shows great potential in the research of prostate cancer treatment (145–147). For example, in a clinical study, the gene of PSA was loaded into adenovirus type 5 (Ad5) to make a viral vaccine to treat patients with mCRPC. Most of the patients detected anti-PSA immune response, especially the increase in the proportion of PSA-specific T cells (148). Of course, the use of viral vaccines may be accompanied by adverse events associated with them (149), including mild adverse reactions (local pain, swelling or induration and systemic fever) and severe adverse reactions (thrombosis and/or allergies) (150, 151). However, with the application of covid-19 adenovirus vaccine, biosafety problems may be forced to be solved, which may promote the development of viral vaccines.

Bacterial immunotherapy for prostate cancer: most of the current research is limited to gut bacteria, and there are few studies for prostate cancer bacteria (152). But prostate bacteria are indispensable in prostate cancer immunotherapy. In a recent animal experiment, it was discovered that the facultative anaerobe Escherichia coli could specifically produce TNF-α in mouse tumors (153). Although TNF-α can induce tumor cell apoptosis (154), it has been abandoned as a cancer treatment due to systemic side effects (155). Research on bacterial immunotherapy may now avoid this side effect and allow TNF-α to be reapplied to cancer therapy.

### Targeted Therapy of Prostate Cancer by Microorganisms

The use of microorganisms to prevent and treat prostate cancer may be a popular treatment strategy in the future. Based on the characteristic that specific microorganisms can deliver exogenous genes to prostate cancer cells to interfere with the proliferation of prostate cancer cells (67), or through the microorganisms themselves or their metabolites to activate some protein kinases to regulate the apoptosis of prostate cancer cells (156), in order to achieve the purpose of treating prostate cancer. At present, the microorganisms that can potentially become targeted therapies are mainly non-pathogenic bacteria and viruses.

Because the tumor microenvironment is usually accompanied by hypoxia and insufficient blood supply, conventional treatments such as chemotherapy and immunotherapy often cannot achieve the desired effect (157), and hypoxia is closely related to local biochemical recurrence. Therefore, the combined or single use of bacteria targeting therapy for prostate cancer is one of the most feasible ways in the future, especially for anaerobes or facultative anaerobes. Salmonella typhimurium, which belongs to anaerobic bacteria, can express green fluorescent protein (GFP) and induce the death of PC-3, LNCaP, and DU-145 prostate cancer cells through different mechanisms (158). Serratia marcescens, which belongs to facultative anaerobes, can inhibit the growth of prostate cancer cells by down-regulating IAP family inhibitors XIAP, CIAP-1, and CIAP-2, activating caspase-9 and caspase-3, and this is accompanied by the degradation of poly-ADP-ribose polymerase (156). The research and discovery of more specific bacteria will lay the foundation for the targeted treatment of prostate cancer.

An oncolytic virus is a kind of tumor-killing virus with replication ability, whether naturally occurring or genetically engineered, that can specifically infect and dissolve cancer cells without damaging normal cells. Some of them can bind to tumor cells through abnormally expressed or up-regulated surface receptors in tumor cells, while others can only replicate in tumor cells with defective signal pathways due to the loss of virulence genes (159). Furthermore, some oncolytic viruses enhance the immune response and attract more immune cells, allowing leftover cancer cells to be killed (160). Mammalian orthovirus (MRV) is one of the oncolytic viruses targeting tumor cells. It can down-regulate HIF-1α and induce apoptosis of prostate cancer cells under hypoxia by activating caspase-8 and caspase-9 (161). MRV infection can reduce the activity of activated Akt and AR proteins and the expression of PSA in prostate cancer cells, so it may inhibit the progression of prostate cancer to CRPC (162). MicroRNAs were also inserted into the 3’ untranslated region (3’-UTR) of the HSV-1 basic viral gene, allowing the virus to selectively target cancer cells and reduce toxicity to normal tissues (163). In addition, it is also a suitable way to induce apoptosis of prostate cancer cells by inserting different protective antigen genes to regulate the apoptosis mechanism, including Caspase family, IAPs family, and Bcl-2 family (164, 165). Because the recombinant genome does not enter the chromosome, there is no risk of insertion mutation (166, 167). At the same time, considering that viral therapy is mainly aimed at anoxic sites or extensive metastatic sites with high resistance to traditional therapy, some studies have also focused on the characteristics of mesenchymal stem cells differentiating into various cells (such as macrophages). With the help of new techniques such as ultrasound targeting to transport the virus to the target cells, this may greatly improve the effectiveness of tumor therapy and reduce possible side effects (168, 169). Although most clinical trials on adenovirus-mediated gene therapy and viral therapy have shown good anti-tumor effects, this potential treatment is under consideration because of possible biosafety problems.

Microbial-based prostate cancer prevention and treatment strategies may run through the whole course of treatment in the future, making up for the shortcomings of traditional prostate cancer treatment methods, such as prostate cancer diagnosis, tumor resection, chemotherapy, radiotherapy, endocrine therapy, and so on. It can greatly improve the prognosis and prognosis of prostate cancer.

## Conclusions and Future Perspectives

Based on previous studies, it is not difficult to find that the microflora in the prostate microenvironment is constantly emphasized in tumorigenesis, invasion, metastasis, and biochemical recurrence. A variety of biological mechanisms regulated by various microorganisms and their metabolites may be involved in the process of prostate cancer. These processes include the well-known indirect effects mediated by immune surveillance and direct effects in the early stages of research. These mechanisms not only regulate the transformation of prostate epithelial cells from benign to malignant, but also promote or inhibit prostate cancer. This indicates that it is a future research trend to explore the etiology and mechanism of prostate cancer in the field of microbiology.

At present, there are obvious defects in the treatment of prostate cancer; that is, the residual cancer cannot be completely eliminated, and there is a high risk of local biochemical recurrence after the treatment of prostate cancer. Microbial immunotherapy and targeted therapy can make up for the limitations of traditional therapy. The single or combined application of medical methods in the field of microbiology may herald the dawn of cancer patients. In the future, we should conduct a more in-depth study of the microflora of prostate cancer, explore its potential role in the prostate microenvironment, and continue to carry out microbial tumor therapy, which has positive significance for the prevention, early diagnosis, treatment, and prognosis of prostate cancer.

There are potential biosafety problems in the use of microorganisms, especially recombinant viruses or bacteria that are widespread in laboratories. In clinical trials, it is often accompanied by some side effects. However, it is undeniable that with the application of live attenuated measles vaccine, live attenuated hepatitis A vaccine, COVID-19 inactivated vaccine, and other viral vaccines, these biosafety problems will be solved.

## Author Contributions

KT conceptualized the review, analyzed the data, and helped to write the manuscript. BC, WZ, SX, TH, WL, YY, JH, and JY helped to write the manuscript and prepared the figures. All authors contributed to the article and approved the submitted version.

## Funding

This study was supported by the National Natural Science Fund of China (81660263), Science and Technology Fund Project of Guizhou Health Commission. (gzwkj2021-211), and Doctoral Fund of Affiliated Hospital of Guiyang Medical College, Guizhou Province, China (C-2012-6).

## Conflict of Interest

The authors declare that the research was conducted in the absence of any commercial or financial relationships that could be construed as a potential conflict of interest.

## Publisher’s Note

All claims expressed in this article are solely those of the authors and do not necessarily represent those of their affiliated organizations, or those of the publisher, the editors and the reviewers. Any product that may be evaluated in this article, or claim that may be made by its manufacturer, is not guaranteed or endorsed by the publisher.
